# Supplementary material for: Duplex PCR assay to determine sex and mating status of Ixodes scapularis (Acari: Ixodidae), vector of the Lyme disease pathogen
Source: J Med Entomol. 2025 May 7;62(4):800–7. doi: 10.1093/jme/tjaf043 (PMC12271729; doi:10.1093/jme/tjaf043)
Supplement: tjaf043_suppl_Supplementary_Figures_S1-S5 [file tjaf043_suppl_supplementary_figures_s1-s5.pdf]

Duplex PCR assay to determine sex and mating status of *Ixodes scapularis* (Acari: Ixodidae), vector of the Lyme disease pathogen

Isobel Ronai[1,2]*, Julia C. Frederick[3,4]*, Alec T. Thompson[4,5], Prisha Sharma[3], Michael J. Yabsley[4,5,6], Utpal Pal[7,8], Cassandra G. Extavour[1,2,9] and Travis C. Glenn[3,4,10]

Supplementary materials

Table S1. Resequencing sample information and sequencing statistics.

Table S2. Triple-enzyme restriction-site associated sequencing sample information and sequencing statistics.

Table S3. Two male-specific loci identified in the triple-enzyme restriction-site associated sequencing dataset.

Table S4. Male and females accurately sexed using the molecular sexing method from colony samples and field samples.

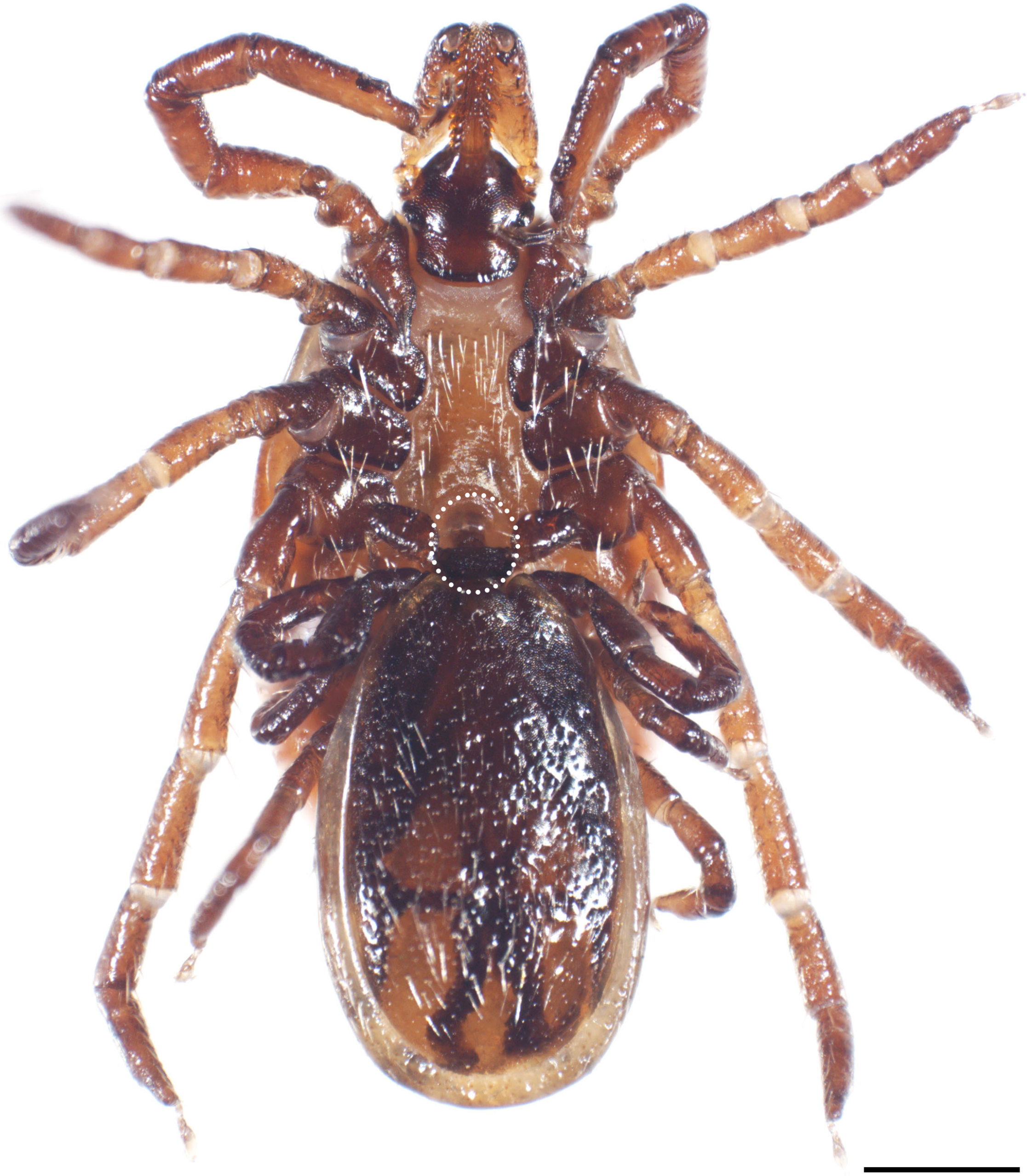

Figure S1. *Ixodes scapularis* mating. Ventral view of an adult male with his hypostome inserted into the adult female's genital aperture (circle) on her dorsal side. Anterior is up and scale bar represents 0.5 mm.

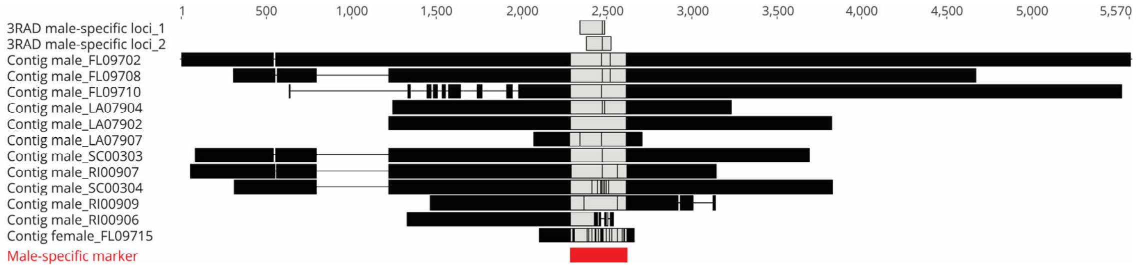

Figure S2. Identification of an *Ixodes scapularis* male-specific genomic region. Multiple sequence alignment: two overlapping male-specific loci from the 3RAD dataset; 11 contigs from male resequencing assemblies, with a high confidence match to the two male-specific loci; one 552 bp contig from a female resequencing assembly, with a low confidence match to the two male-specific loci; and the identified male-specific marker (red). The site position of the alignment is in bp. Nucleotide positions that match the male-specific marker (reference sequence) are colored grey, whereas positions that disagree with the male-specific marker or are outside the marker are colored black.

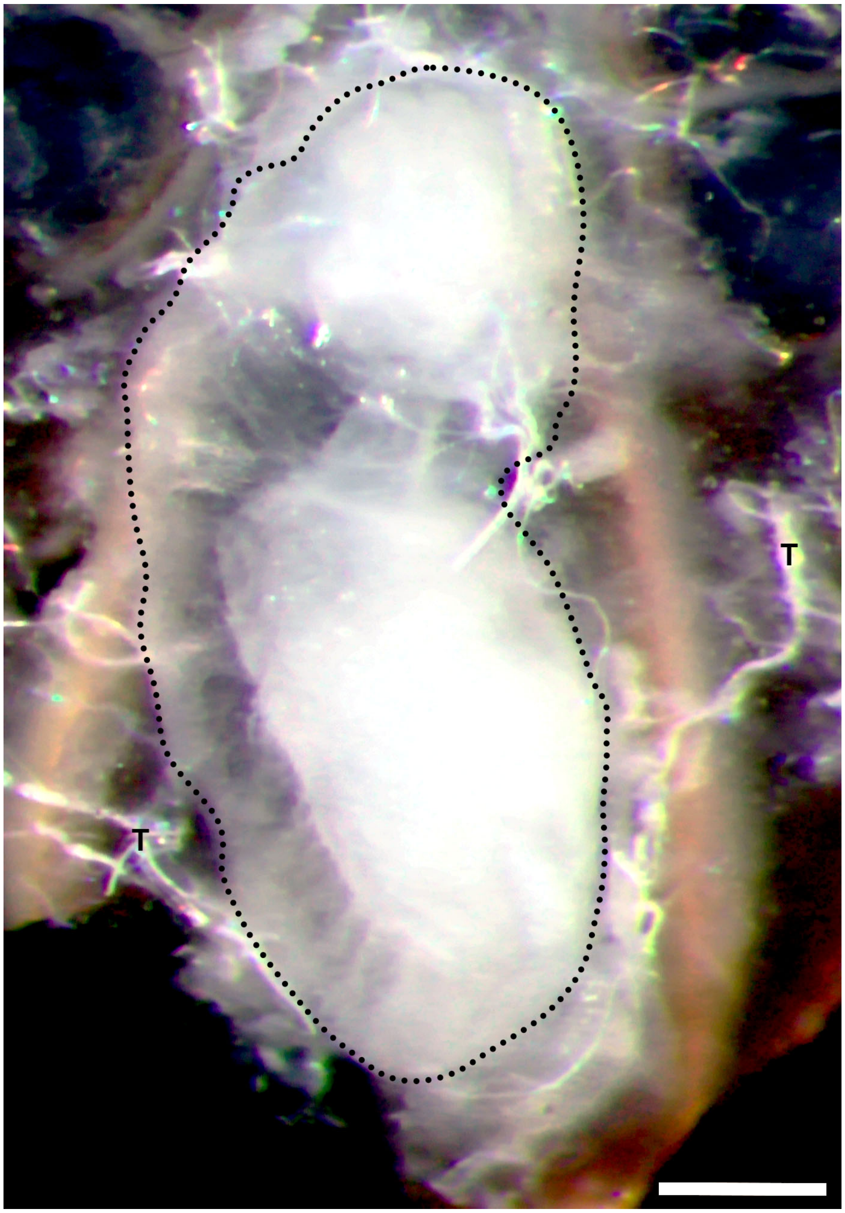

Figure S3. Endospermatophore (dotted line) inside a dissected adult female *Ixodes scapularis* that was paired in the laboratory mating assay. Trachea (T). Dorsal view, anterior is up and scale bar represents 0.5 mm.

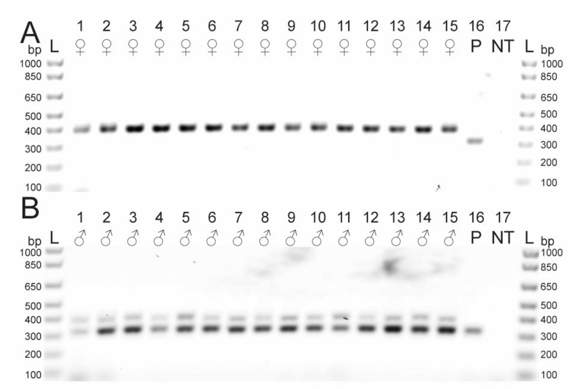

Figure S4. Adult *Ixodes scapularis* from the Oklahoma State University colony using molecular sexing method (duplex PCR) visualized on an agarose gel. Primers described in Table 1. All female samples have the expected single 406 bp band. All male samples have the expected double band at 406 bp and 326 bp. The plasmid positive control has the expected single band at 326 bp. Fifteen samples (Lane 1-15), plasmid (P) positive control (Lane 16), no template (NT) negative control (Lane 17). (A) Females used as template. (B) Males used as template.

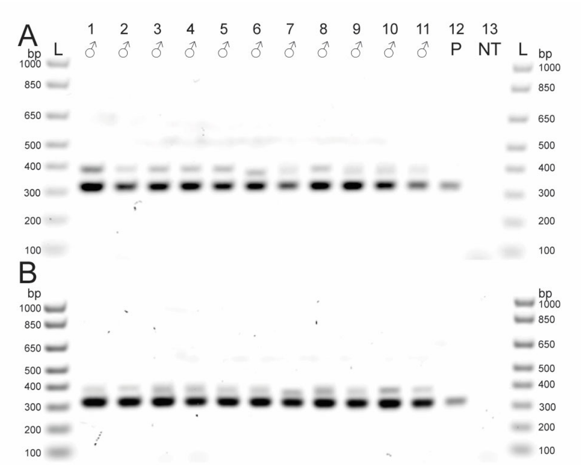

Figure S5. Adult male *Ixodes scapularis* that were paired with females in the laboratory compared with males that were never paired, using the molecular sexing method (duplex PCR) visualized on an agarose gel. Primers described in Table 1. All samples have the expected double band at 406 bp and 326 bp. The plasmid positive control has the expected single band at 326 bp. Eleven males (Lane 1-11), plasmid (P) positive control (Lane 12) and no template (NT) negative control (Lane 13). (A) Paired males used as template. B) Unpaired males used as template.
